# Clinical forensic height measurements on injured people using a multi camera device for 3D documentation

**DOI:** 10.1007/s12024-020-00282-9

**Published:** 2020-07-12

**Authors:** Till Sieberth, Lars C Ebert, Simon Gentile, Barbara Fliss

**Affiliations:** 1 3D Zentrum Zurich, University of Zurich, Winterthurerstrasse 190/52, 8057 Zürich, Switzerland; 2 Institute of Forensic Medicine, University of Zurich, Winterthurerstrasse 190/52, 8057 Zürich, Switzerland

**Keywords:** Photogrammetry, Forensics, Injury documentation, Photobox, Digitization, 3D documentation

## Abstract

Documenting the existence, size, position and shape of injuries is an important part of medical forensic examinations. In the photography of an injury, the documentation is limited to an approximation of size and position of the injury based on a ruler included in the image. The documentation of injuries can be improved with photogrammetry, which allows the creation of scaled 3D models of an injury that can be used to not only document and visualize the injury but also to match the injury with an injury-causing object. In this paper, the multicamera device “Botscan” was used to perform 3D whole-body documentation and measure the positions of injuries. A major advantage of 3D whole-body documentation compared to photography is that the former can be performed at a later stage of the investigation. This makes the whole-body 3D documentation of injuries an important tool for re-examination.

**Electronic supplementary material:**

The online version of this article (10.1007/s12024-020-00282-9) contains supplementary material, which is available to authorized users.

## Introduction

The documentation of incidents is an important procedure in forensics [1–4]. In forensic medicine, measurements of body height and injury positions are essential for the description and evaluation of an injury [5, 6]. It has been shown that injuries at different heights on the body of a pedestrian involved in a traffic collision allow investigators to make assumptions about the vehicle [1, 7]. In this article, we used the multicamera device Botscan© (botspot GmbH, Berlin, Germany) (Photobox) to measure and document injury positions on living people.

**Fig. 1:**
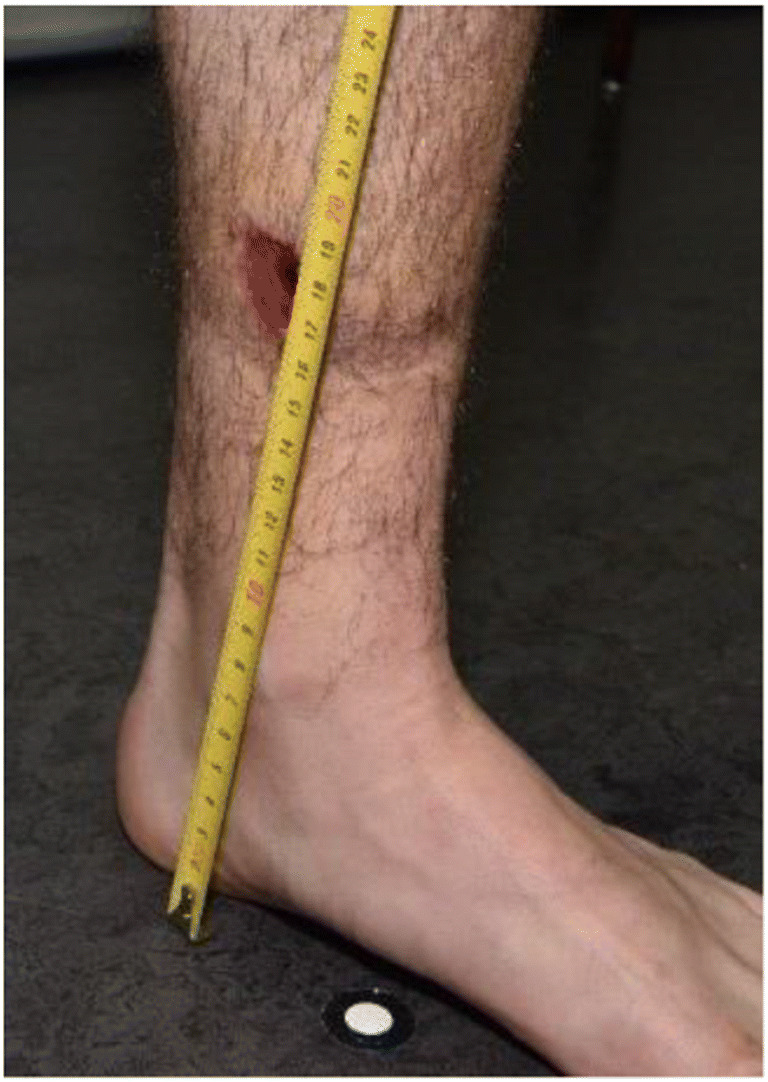
Tape measurement for height localization in forensic medicine on an example of a leg injury

In forensic medicine, the measurements of injury shape and positions often depend on the description of the examiner, who can only perform measurements during and after the examination based on the documentation that was performed, such as body schemes and photographs. An extensive literature search revealed no standard procedure describing how to accurately describe and measure the position of injuries. However, several references stated that the positions of injuries should be documented [1, 4, 6–8]. According to internal guidelines of the Zurich Institute of Forensic Medicine, in cases of traffic collisions, the heights of injuries are measured with a tape measure drawn out from the floor to the injury (Fig. 1).

For an accurate measurement, a tape measure or folding ruler should represent a straight, vertical line from the ground to the injury. This is problematic because holding the tape or ruler by hand often results in a roughly estimated vertical line. Furthermore, the tape often cannot be held with the necessary pressure, as it is difficult to affix the tape on the ground and simultaneously hold it at the necessary height. Third, the reading must be made orthogonally to the tape, which can be difficult for the person already holding it. It can also happen that the tape may also be slightly bent along the body to achieve the necessary stability, which does not result in a straight line from the ground but rather a pathway along the bent tape. In addition to executing the procedure, the measurement also has to be documented. In an optimal case, the measurement is not just written down but also documented with a photo showing the position of the injury along the tape and on the body. Additionally, the documentation should be carried out swiftly for the benefit of all involved parties.

### Requirements

To improve this procedure, three-dimensional (3D) documentation was tested. 3D scanning of objects and scenes is a well-established procedure [9–11]. We considered the following requirements for the documentation of injuries:

1. Documenting detail: A detailed documentation of the shape, color and size of the injury is of interest. For body examinations, from the view of forensic medicine, it is desirable to document the injury appearance together with a scale to provide an understanding of the injury size. This is normally achieved by taking 2D photos of the injury with a scale in photo.
2. Localization of injury: In addition to the injury itself, the location of the injury should be documented [6, 8]. It is common for the height above the sole of the foot and offset of the body axis to be recorded; however, there are no standard procedures on how to perform these measurements. The accuracy requirements depend on the use of the measurements. It must be considered that the height of a human varies throughout the day [12–14]. Diurnal variation can be up to a few centimeters each day [12]. This variation mostly influences measurements on the upper body above the hip and is proportionally smaller for shorter measurements.
3. Swiftness and ease of documentation: The documentation and recording should be robust, standardized, user-independent and objective. It should be fast and easy. Injured persons might not be able to maintain a static position for several seconds or even minutes due to pain. Perpetrators, on the other hand, might not be willing to cooperate, thus affecting the accuracy of measurements.

To achieve these requirements, several systems were considered. Common methods for documentation include laser scanning and surface scanners [15, 16]. However, both are problematic, as they do not acquire color information and document only a limited part of the body [17–20].

The device used for this paper was the multicamera device Botscan [21]. The so-called Photobox is a device containing 70 cameras and several light panels. The cameras are spread over 12 segments, forming a circle around a center platform to ensure complete coverage of an object on the center platform [21]. The settings of all 70 cameras can be controlled with the software Smart Shooter 3 (Kuvacode OY, Kerava, Finland) [22] to allow adequately illuminated images and simultaneous camera release with the push of a single button [21].

The first test showed that the Photobox data could be used to measure the size of injuries accurately; however, accurate representations of injury shape, color and type have not yet been evaluated [23, 24]. Whether the absolute position of an injury on the body can be established, or if measurement of the height above the sole of the foot can be performed, have also not been evaluated.

The aim of this study was to evaluate whether 3D documentation performed with the Photobox allows the measurement of injury heights. Measurements made with this device were compared to measurements made by the current tape measure method to evaluate whether the Photobox can be used as an alternative.

## Method

This study used a mannequin equipped with injury stickers measured with tape and compared them to measurements performed with a 3D model created using the Photobox [23]. Furthermore, two real cases were included, and these measurements were performed as part of the case documentation. The measurements of the two injured persons taken during the medical examination were compared to the measurements taken using the 3D model.

### Test subjects

The mannequin (Pujiang Xufeng Hanger Co. Ltd., Zhejiang, China) was equipped with over 40 injury stickers (Tinsley Transfers, San Fernando, CA, USA) [23]. However, only 22 injury stickers on the torso and legs were used for the study, as the arms and head can move around freely, especially during transport from the examination area to the scanning area. To avoid interobserver errors and to test the system rather than the observers, the injuries were clearly marked with small points to ensure that both the medical examiner and technical personnel measured the same location.

The included cases involved two males who each claimed to have been injured by a car driving into them. The medical examiner documented six injuries on each person, which were on the legs, torso and head. While the points of measurement were clearly marked on the mannequin, the injuries on the persons could not be marked.

### Medical examination

The measurement of the injury heights was performed in accordance with the institute’s internal guidelines. A tape measure was used to measure the heights of the injuries from the floor to the injury. This was performed for all injuries on the legs and torso but not for the arms, as the arms are usually not the initial contact point during a collision. The localization measurements were performed to a central point of the injuries with a diameter less than two centimeters, and the heights to the top and bottom of larger injuries were measured. For documentation purposes, each measurement was photographed, and notes and sketches were made on a template of the body outline. After successful measurements in the medical examination, the mannequin and the human subjects were documented using the Photobox.

### Scan procedure

To scan the mannequin, it was placed onto the scanning platform, and the cameras were released. The positions of the mannequin’s legs and torso were not modified, as they were fixed in position.

Furthermore, the injuries of the two persons involved in traffic collisions were documented. A few aspects have to be considered to scan a person for all necessary information. Clothing, hair and accessories might be some of the most important aspects to consider. In documenting injuries, it is necessary to see the actual injuries, which means that clothing might interfere with documentation and need to be removed. Hair can also be an interference, and depending on the severity of the injury, it might be necessary to either move the hair that covers the injury or, in severe cases, cut or shave the hair to allow proper documentation for both 2D and 3D documentation. People are typically documented without shoes or accessories, as these items might influence the height of documented injuries or affect the visibility of some body parts. A subsequent addition of the shoe height could be performed but was not necessary for this study.

**Fig. 2:**
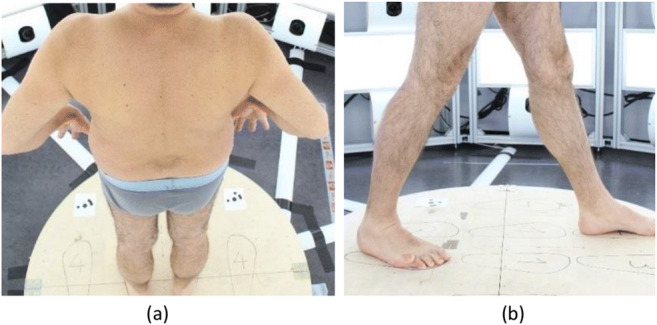
Person standing in the Photobox in a standard posture (a) and stepping forward (b) for easier documentation of injuries between the legs

Another important aspect is the body posture that the person should have during a scan. First, it should be possible for every person to assume the required posture, and the posture should be easy to explain. For forensic 3D reconstruction and body height measurements, a suitable posture entails the person standing up straight and facing forward with the feet shoulder-width apart. The hands should be at the side of the body, the thumb should mark the top of the hip (anterior superior iliac spine), and the index finger should point down the leg with the fingers spread. The elbow should be spread outwards (Fig. 2a).

With this posture, the shoulder, elbow and wrist are visible. The hip is marked with the thumb, and the knee and ankle should be visible when the person is wearing shorts. Although this posture has been shown to be efficient for forensic 3D reconstruction, it does cause some problems in the documentation of injuries. In this posture, some cameras may not be able to document injuries on the inside of the legs, which causes problems with the photogrammetric reconstruction. For this purpose, additional foot positions were tested, such as stepping forward with either leg (Fig. 2b) and standing with both legs wider than shoulder width to enable more cameras to see the inside of the legs. This posture was applied with the traffic accident victims but could not be applied with the mannequins due to the fixed posture of the mannequin.

To ensure that the 3D reconstruction was scaled correctly, reference scale bars were included in the scene. Several automatically detectable targets were placed all around the interior and on the platform, and the distance between them was measured with a tape measure.

### Processing

**Fig. 3:**
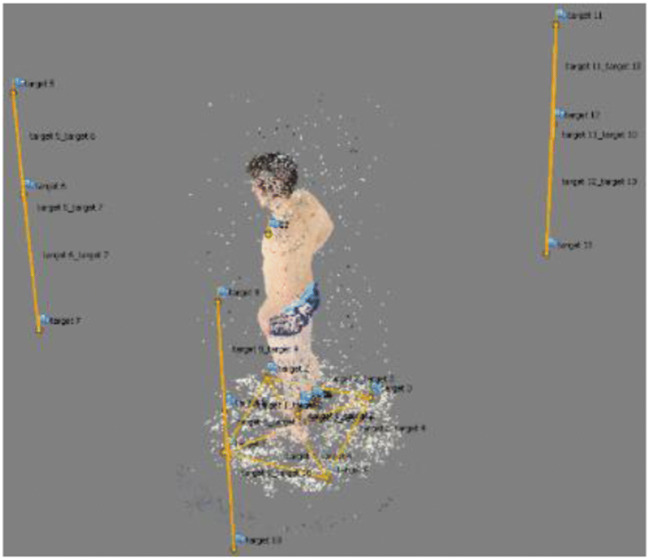
Coarse point cloud of a person (center) with targets (yellow dots) and scale bars (yellow connection lines between dots)

**Fig. 4:**
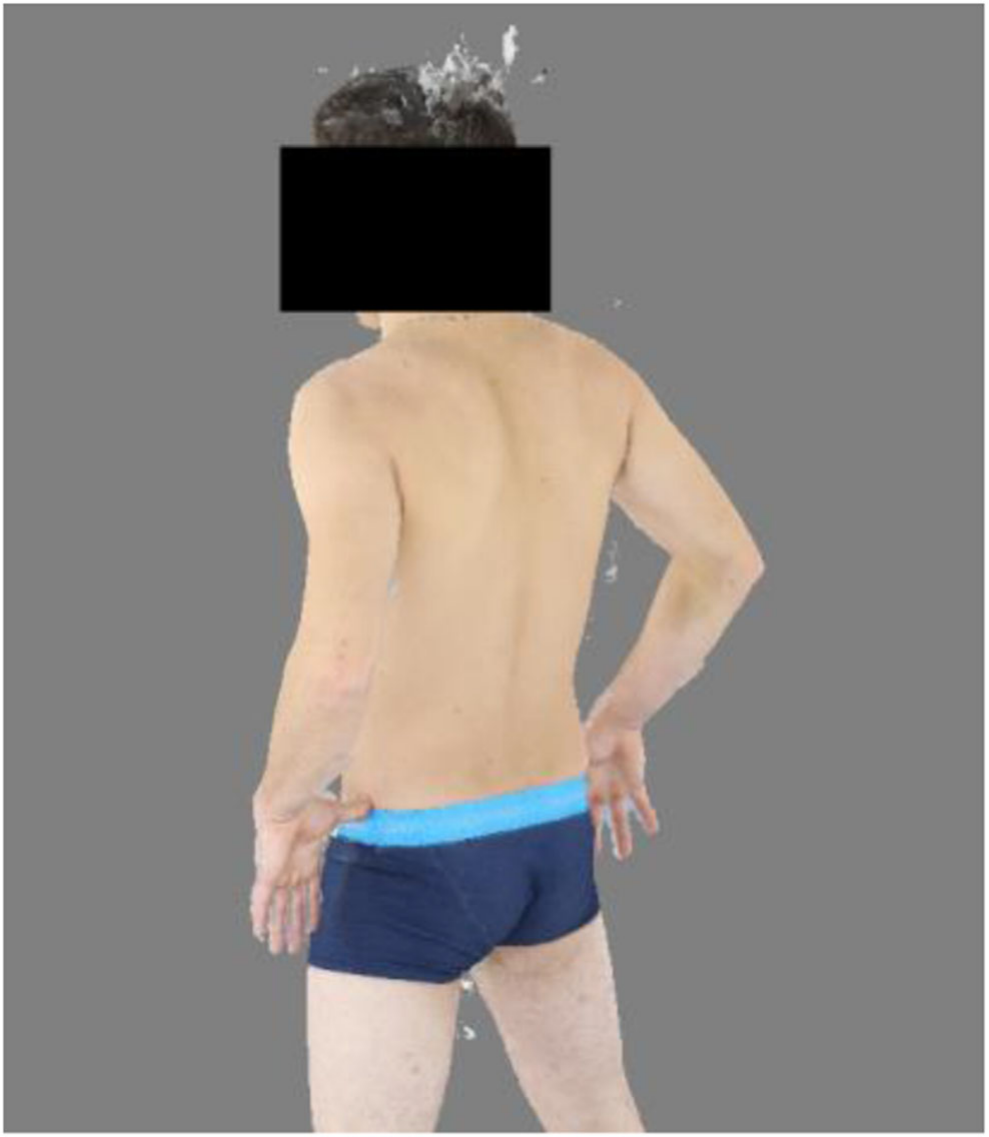
Textured model. Artifacts can be seen at the head, between the legs and under the right arm. However, these factors do not influence the subsequent processing

For the calculation of the 3D model, the software Agisoft PhotoScan (v1.2.6 build 2834) [25] was used to process the images. The first step conducted with the software was to automatically detect the targets in the images. After the completion of this step, it was possible to enter the length of the scale bars and reference the previously detected targets so that the program could scale the model correctly (Fig. 3). After these preparatory steps, some user input was performed to define the settings for the photogrammetric calculations (see Appendix). The calculation itself was mostly automatic and only required some postprocessing to clean up artifacts. Ultimately, a textured and scaled 3D model was generated that was used for further processing (Fig. 4).

### Measurements on the 3D model

The 3D models were subsequently used to measure the heights of the injuries. The measurements were also performed in Agisoft PhotoScan. Based on the ability of Agisoft Photoscan to include user-composed Python scripts, a script was written that calculated the shortest distance between points marked on the 3D model to a predefined plane (see Appendix). The plane was defined by four targets placed on the central platform on which the mannequin/person was standing during the scan procedure. Then, each injury was marked on the 3D model according to the medical examination. Injuries with a diameter smaller than two centimeters were marked in the center, while the tops and bottoms of larger injuries were marked. Then, the script automatically calculated the distance between injury points and the ground plane.

## Results

First, the coordinates of the scale bar targets were used to check whether the scaling of the 3D models was correct. For this purpose, the distance between the targets of the scale bars was calculated and compared to the known distances of the scales. It was found that the maximum discrepancy was 15.6 mm and that the average discrepancy was 6.9 mm. For additional control, the distances between the plane and each of the four targets on the center platform were calculated, which should be close to zero. The largest distance was 3 mm when the left foot was forward (Table 1). On average, the distance was 1.2 mm (Table 2).

**Table 1:** Distance between ground points and the mathematical plane that defined the floor for the measurements in Agisoft

Foot Position	Body Examination		
	Mannequin [mm] (*n* = 4)	Person 1 [mm] (n = 4)	Person 2 [mm] (n = 4)
Straight	0 ± 0.4	0.4 ± 0.01	0.6 ± 0.02
Left Foot forward		1.0 ± 0.03	2.9 ± 0.08
Right Foot forward		0.9 ± 0.03	1.6 ± 0.05
Spread legs		1.4 ± 0.04	1.1 ± 0.03

**Table 2:** Distance between targets and the ground plane when the left foot is forward (Fig. 3). Negative values indicate that the target is below the plane

	Position of Target	Distance [mm]
Target 1	Left in front	−2.8
Target 2	Right in front	2.8
Target 3	Right behind	−3.0
Target 4	Left behind	2.9

After it was established that the scale was correct, the measurements performed for the injuries were compared to one another. For the mannequin, the average discrepancy between the medical examination and the 3D model for 22 injuries with top and bottom measurements was −6 mm (±5 mm). The measurements performed with the 3D model were larger than the measurements performed during the medical examination (Table 3).

**Table 3:** Discrepancies of measurements between the 3D model of the mannequin and measurements made with tape

Injury Location		Tape Measurement [m]		Photobox [m]		Discrepancy [m]	
	Top	Bottom	Top	Bottom	Top	Bottom	
Torso Front	Stab Injury	1.38	1.36	1.376	1.361	−0.004	0.001
	Stab Injury	1.38	1.355	1.388	1.360	0.008	0.005
	Bruise	1.25	1.22	1.256	1.215	0.006	−0.005
	Abrasion	1.28	1.24	1.289	1.252	0.009	0.012
	Bruise	1.14	1.12	1.147	1.131	0.007	0.011
	Stab Injury	1.17	1.17	1.176	1.168	0.006	−0.002
Torso Back	Abrasion	1.34	1.285	1.349	1.293	0.009	0.008
	Abrasion	1.195	1.13	1.203	1.140	0.008	0.010
	Stab Injury	1.13	1.12	1.135	1.121	0.005	0.001
	Stab Injury	1.34	1.29	1.339	1.302	−0.001	0.012
	Abrasion	1.47	1.41	1.470	1.408	−0.000	−0.002
	Stab Injury	1.53	1.525	1.537	1.521	0.007	−0.004
Left Leg	Stab Injury	0.73	0.69	0.739	0.701	0.009	0.011
	Abrasion	0.57	0.53	0.578	0.537	0.008	0.007
	Bruise	0.5	0.475	0.502	0.482	0.002	0.007
	Abrasion	0.27	0.23	0.279	0.236	0.009	0.006
	Abrasion	0.79	0.765	0.799	0.775	0.009	0.010
Right Leg	Stab Injury	0.69	0.67	0.694	0.675	0.004	0.005
	Bruise	0.4	0.37	0.409	0.379	0.009	0.009
	Stab Injury	0.66	0.625	0.667	0.633	0.007	0.008
	Bruise	0.825	0.81	0.831	0.811	0.006	0.001
	Stab Injury	0.44	0.415	0.445	0.427	0.005	0.012
Average discrepancy [mm] (*n* = 44)						6	
Standard deviation [mm] (n = 44)						5	

For the real cases, it was found that the measurements were shorter when the person was stepping forward than when the person was standing up straight (Tables 4 and 5).

**Table 4:** Discrepancies between measurements based on the Photobox models and measurements made with tape on Person 1

			Photobox Model measurement discrepancies			
Injury position		Tape Measurement [m]	Standing Straight [m]	Left Foot Forward [m]	Right Foot Forward [m]	Legs Spread [m]
Chest	Top	1.24	0.016	−0.022	−0.031	−0.005
	Bottom	1.23	0.020	−0.019	−0.028	−0.001
Left Calf	Top	0.19	0.000	−0.009	−0.009	−0.011
	Bottom	0.17	−0.016	−0.018	−0.027	−0.023
Right Ankle	Top	0.12	−0.020	−0.021	−0.022	−0.016
	Bottom	0.1	−0.010	−0.012	−0.011	−0.006
Average discrepancy [mm] (*n* = 6)			−2	−17	−21	−10
Standard deviation [mm] (n = 6)			15	5	8	7

**Table 5:** Discrepancies between measurements based on the Photobox models and measurements made with tape on Person 2

Injury position		Tape measurement [m]	Photobox Model measurement discrepancies			
			Standing Straight [m]	Left Foot Forward [m]	Right Foot Forward [m]	Legs Spread [m]
Left Knee	Front	0.48	−0.019	−0.024	−0.057	−0.026
	Left	0.49	−0.013	−0.022	−0.055	−0.018
Left Eye	Bottom	1.63	0.030	−0.011	−0.008	0.030
	Top	1.65	0.030	−0.011	−0.010	0.027
Forehead		1.7	−0.011	−0.056	−0.050	−0.013
Average discrepancy [mm] (*n* = 5)			3	−25	−36	0
Standard deviation [mm] (n = 5)			22	16	22	24

**Fig. 5:**
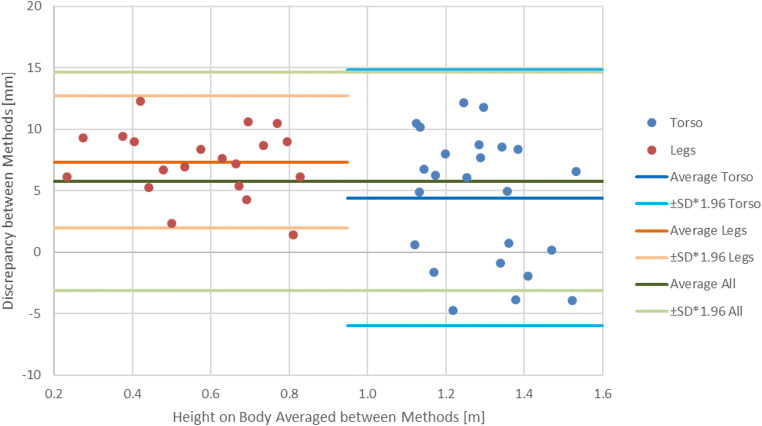
Bland-Altman plot for measurements performed on the mannequin with the average for all injuries and separately for the legs and torso

**Fig. 6:**
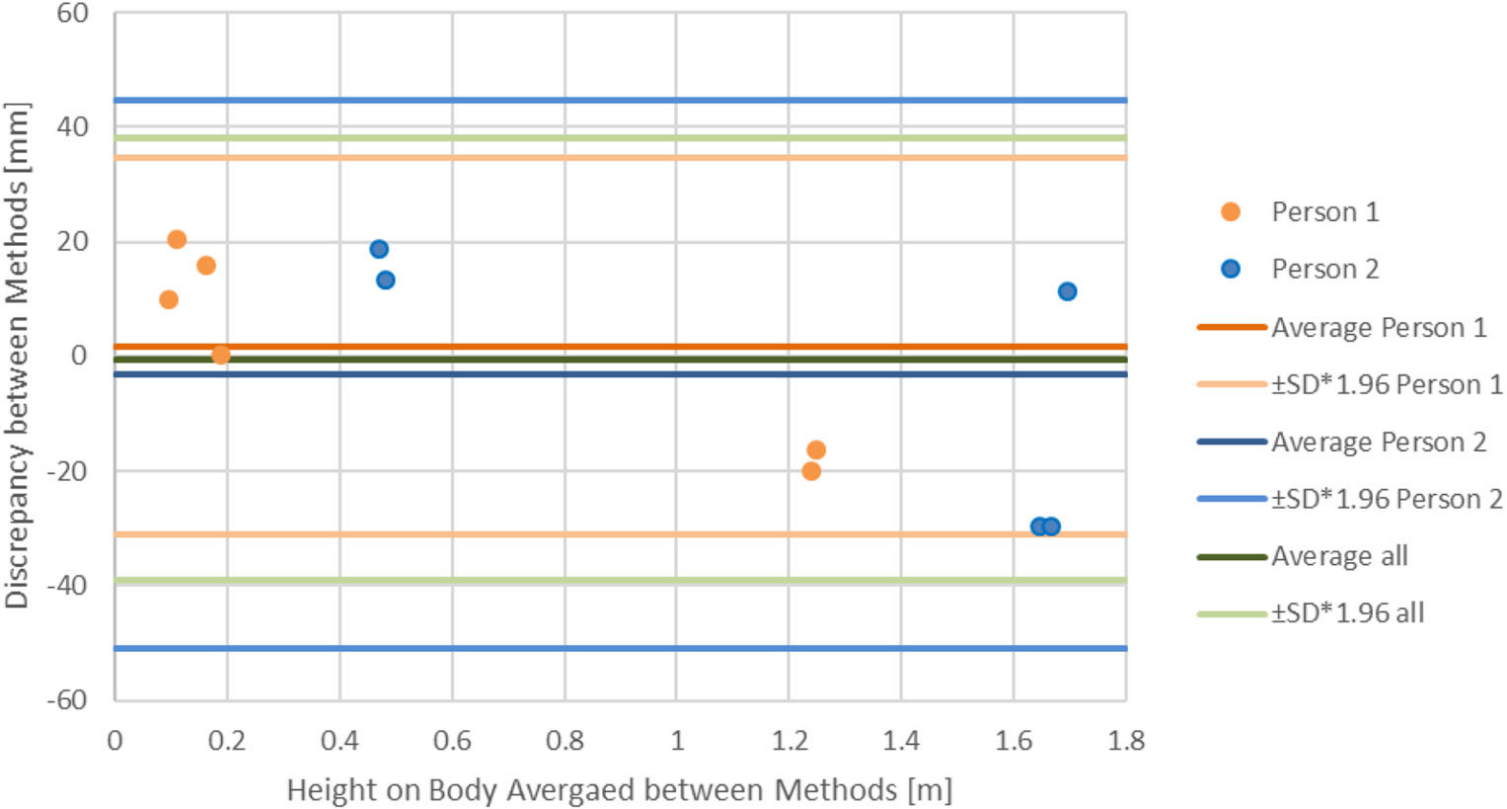
Bland-Altman plot for measurements performed in the real cases with the average for all injuries and separately for each person

The Bland-Altmann plot for the mannequin shows that both methods yielded comparable results (Fig. 5). For the injuries on the legs, the variance in the discrepancies was smaller than the variance for injuries on the torso; overall, the measurements performed with the 3D model were systematically longer than the measurements performed during the medical examination. For the real cases, the Bland-Altman plot showed that the measurements were similar but had a larger variance than the measurements on the mannequin (Fig. 6).

## Discussion

In this article, we presented and tested the Photobox for the 3D documentation of injury positions and compared the results to measurements performed with tape measures during a medical examination procedure.

It was shown that the Photobox data can be used to establish the location of injuries. The variance of 5 mm for the mannequin is in an acceptable range considering the many factors that could negatively influence the measurements of a stable object. The main problems are that during the medical examination, it can be difficult to establish and reach the correct foot that is planted on the floor in a perpendicular position and draw out the tape measure to the injury in a straight vertical line. Furthermore, taking a vertical reading on the tape measure while ensuring that the tape is affixed to both the floor and the injury is difficult. Additionally, the point of measurement may not be defined and might differ between physicians for the same injury. Without this definition, the measurement is dependent on the physician, and if the process of measurement is changed in the future, the measurements cannot be repeated. With Photobox data, however, measurements can be repeated for injuries even when the measurement system changes. While the Photobox appears to be an effective tool for injury documentation, the processing of the data requires technical knowledge and therefore skilled users to perform the subsequent measurements. For the mannequin, the injuries were clearly marked with dots at the upper and lower boundary. These dots were used by the physician and for measuring on the 3D model, allowing comparison of the measurements independent of having an undefined injury boundary.

**Fig. 7:**
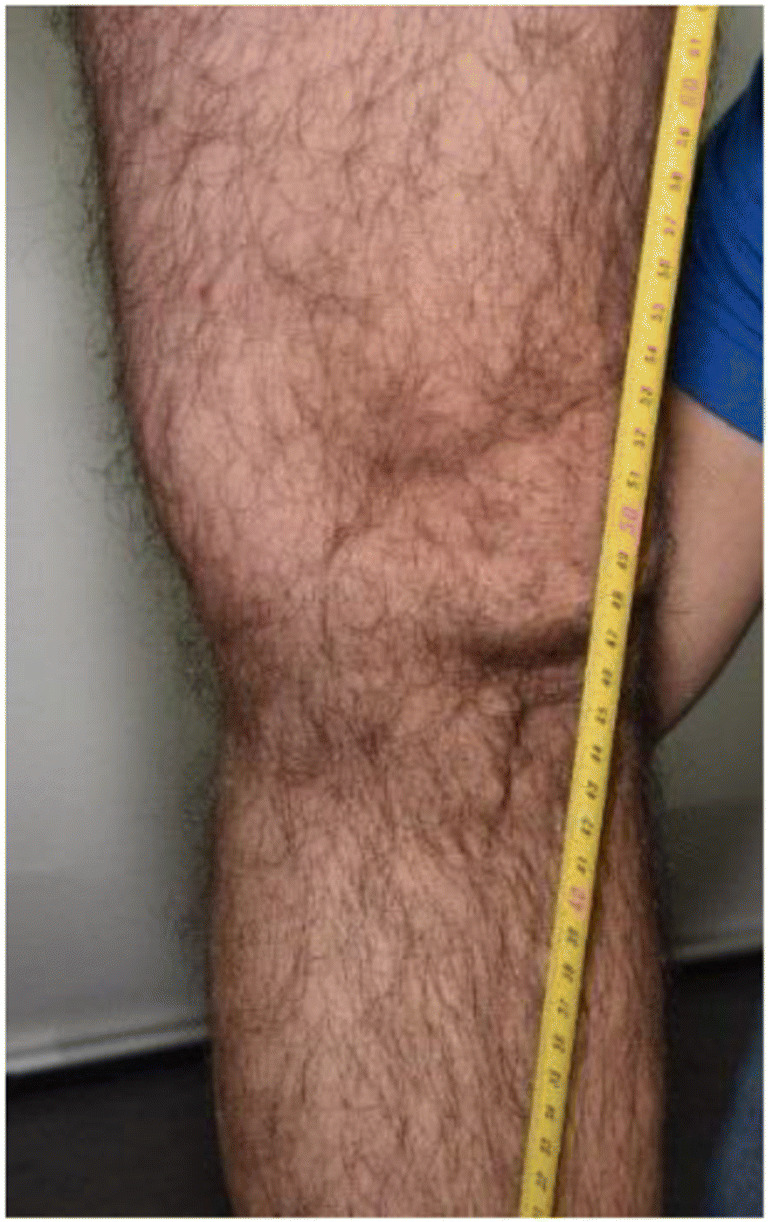
Photography for the documentation of a turgor. However, the turgor is visible neither on the photograph nor in the 3D model so that a measurement on the 3D model was possible

The real case examples show that there are discrepancies between medical examiner measurement and measurements performed on the 3D models; however, the Bland-Altman plot indicates that the measurement discrepancies are well within a 95% agreement limit. One problem might be that the boundary of injuries is not always well defined, and medical examiners and persons performing 3D measurements might have different understandings of the injury boundary. Another error is human motion, as the persons had to move between the medical examination and 3D documentation, during which they altered their body positions and therefore the measurements. This also suggests that foot sole measurements, independent of the measurement method, can be different from those at the time of the actual event. Another finding with the real case examples was that the center platform moves depending on the foot position and stance, which influences the ground plane and therefore the subsequent measurements. This can be solved by documenting the persons in a defined shoulder width stance. The real case examples also posed another problem for the reassessment of nonvisible injuries, such as swelling. During the medical examination, the physician documented turgor at heights of 0.44–0.54 m, which was visible neither on the 3D model nor on the documentation photograph (Fig. 7).

The Bland-Altman plots show that for both mannequins and real cases, the measurements made with the Photobox are comparable to measurements made during the medical examination. This allows us to state that the Photobox can be used to perform injury documentation and localization. Due to the unavailability of a more accurate method, it is not possible to say whether Photobox or medical examination measurements are more accurate, but we can affirm that both methods yield similar results. However, previous results suggest that the Photobox yields more accurate measurements [23]. Another limitation is that the measurements were only performed by one person for each method. This reflects what would be done in real situations, in which the medical examination is only performed by one medical examiner, and no repeated measurements are performed. This was also done for the measurements on the 3D model to keep both methods comparable, even though with the 3D model, multiple measurements between different persons could have been performed.

In addition to the price, another limitation of the Photobox is that the device is stationary and cannot be moved; thus, it is only possible to document people who can stand up and move to the location of the Photobox. Additionally, physical examination, such as pushing on an injury to differentiate skin reddening vs hemorrhage, is not possible with either the 3D model or the 2D photograph. Despite these drawbacks, the 3D documentation of injured persons not only allows precise measurements of injury location and dimension but could also allow the use of 3D models for subsequent 3D reconstructions, e.g. matching of injury-causing object to injury [26–29].

In particular, the ease of use, the speed and repeatability of measurements and the possibility of reassessing the dimension and localization of injuries provides an advantage for future investigations. Furthermore, photogrammetric images taken by a photographer depend on the experience of the photographer, while the Photobox acquires standardized photos under optimal lighting conditions. Additionally, the Photobox documents the whole body at once in 3D, while forensic images often only show the area of measurement and not the complete tape, thus potentially missing errors that might have occurred during the measurement procedure. However, it still needs to be analyzed whether the Photobox data can be used not only for measurement but also for forensic medical examination of the type and severity of injury.

## Conclusion

The Photobox has been proven to be an effective tool for documenting the location of injuries compared to the current method of foot sole measurements. The use of the multicamera device Photobox has considerable potential for medical forensic examinations and should be further analyzed in future research.

## Key points

1. The multi camera device Photobox is an effective device to document the location of injuries.
2. The Photobox allows for repeated measurements even well after the intial investigation.
3. The Photobox only allows for visual examination of the person, not physical examination.
4. The 3D documentation of injured persons not only allows precise measurements of injury location and dimension but also allows the use of 3D models for subsequent 3D reconstructions.

## Electronic supplementary material

ESM 1 (DOCX 18 kb)
