# Supplementary material for: Clinical forensic height measurements on injured people using a multi camera device for 3D documentation
Source: Forensic Sci Med Pathol. 2020 Jul 12;16(4):586–94. doi: 10.1007/s12024-020-00282-9 (PMC7669809; doi:10.1007/s12024-020-00282-9)
Supplement: Supplementary file 1 — (DOCX 18 kb) [file 12024_2020_282_MOESM1_ESM.docx]

# Appendix

### Settings for Agisoft

Align Photos:

Accuracy: High, Pair Selection: Generic, Key point limit: 40’000, Tie point limit: 4000, Adaptive camera model fitting

Build Dense Cloud:

Quality: High, Depth filtering: Moderate

Build Mesh:

Surface type: Arbitrary, Source data: Dense Cloud, Face count: High, Interpolation Enabled, Point classes: All

Texture:

Mapping mode: Generic, Blending Mode: Mosaic, Texture size/count, 16384x1, No color correction enabled, Hole filling enabled

### Python Code

1. **import** os
2. **import** sys
3. **import** PhotoScan
4. **import** csv
5. **import** math
6. **from** subprocess **import** call
8. **def** projecttolocal(mark, chunk):
9. v=mark.position
10. v.size=4
11. v.w=1
12. T=chunk.transform
13. v_t=T.matrix*v
14. v_t.size=3
15. proj=chunk.crs
16. coord=proj.project(v_t)
17. **return**(coord)
18. **def** FGH(t1, t2, t3):
19. x=[t1[0], t2[0], t3[0]]
20. y=[t1[1], t2[1], t3[1]]
21. z=[t1[2], t2[2], t3[2]]
22. F=((x[1]-x[0])*(y[1]*x[2]-y[2]*x[1])-(x[2]-x[1])*(y[0]*x[1]-y[1]*x[0]))/((z[0]*x[1]-z[1]*x[0])*(y[1]*x[2]-y[2]*x[1])-(z[1]*x[2]-z[2]*x[1])*(y[0]*x[1]-y[1]*x[0]))
23. G=((x[2]-x[1])-F*(z[1]*x[2]-z[2]*x[1]))/(y[1]*x[2]-y[2]*x[1])
24. H=(1-G*y[2]-F*z[2])/x[2]
25. **return**[F, G, H]
26. **def** distance(mark, F, G, H, chunk):
27. **print**("=========================")
28. **print**(F)
29. **print**(G)
30. **print**(H)
31. **print**("")
32. test=0
33. **for** o **in** mark:
34. **if** o.selected:
35. test=1
36. p=projecttolocal(o, chunk)
37. d=-1*(H*p[0]+G*p[1]+F*p[2]-1)/math.sqrt(H*H+G*G+F*F)
38. **print**("{}, {}, {}".format(o.label, d, p))
39. **if** test==0:
40. **print**("Did you select markers for calculation?")
41. **print**("=========================")
42. **def** auto():
43. chunk=PhotoScan.app.document.chunk
44. t1=[0 **for** i **in** range(3)]
45. t2=[0 **for** i **in** range(3)]
46. t3=[0 **for** i **in** range(3)]
47. t4=[0 **for** i **in** range(3)]
48. j=0;
49. **for** i **in** chunk.markers:
50. **if** i.label=="target 1":
51. t=projecttolocal(i, chunk)
52. t1[0]=t[0]
53. t1[1]=t[1]
54. t1[2]=t[2]
55. j=j+1
56. **elif** i.label=="target 2":
57. t=projecttolocal(i, chunk)
58. t2[0]=t[0]
59. t2[1]=t[1]
60. t2[2]=t[2]
61. j=j+1
62. **elif** i.label=="target 3":
63. t=projecttolocal(i, chunk)
64. t3[0]=t[0]
65. t3[1]=t[1]
66. t3[2]=t[2]
67. j=j+1
68. **elif** i.label=="target 4":
69. t=projecttolocal(i, chunk)
70. t4[0]=t[0]
71. t4[1]=t[1]
72. t4[2]=t[2]
73. j=j+1
74. **if** j==4:
75. plane1=FGH(t1, t2, t3)
76. plane2=FGH(t1, t2, t4)
77. plane3=FGH(t1, t3, t4)
78. plane4=FGH(t2, t3, t4)
79. F=(plane1[0]+plane2[0]+plane3[0]+plane4[0])/4
80. G=(plane1[1]+plane2[1]+plane3[1]+plane4[1])/4
81. H=(plane1[2]+plane2[2]+plane3[2]+plane4[2])/4
82. **else**:
83. **print**("Error")
84. distance(chunk.markers, F, G, H, chunk)
85. label="Photobox/Vertical Height"
86. PhotoScan.app.addMenuItem(label, auto)
